# Expression Pattern of Trace Amine-Associated Receptors during Differentiation of Human Pluripotent Stem Cells to Dopaminergic Neurons

**DOI:** 10.3390/ijms242015313

**Published:** 2023-10-18

**Authors:** Nataliia V. Katolikova, Anastasia N. Vaganova, Daria D. Shafranskaya, Evgeniya V. Efimova, Anna B. Malashicheva, Raul R. Gainetdinov

**Affiliations:** 1 Institute of Translational Biomedicine, Saint-Petersburg State University, 199034 Saint-Petersburg, Russiar.gainetdinov@spbu.ru (R.R.G.); 2 Saint-Petersburg University Hospital, Saint-Petersburg State University, 199034 Saint-Petersburg, Russia; 3 Center for Algorithmic Biotechnology, Saint-Petersburg State University, 199034 Saint-Petersburg, Russia; 4 Department of Embryology, Faculty of Biology, Saint-Petersburg State University, 199034 Saint-Petersburg, Russia; a.malashicheva@spbu.ru

**Keywords:** trace amine-associated receptors (TAARs), dopamine (DA), neurogenesis, human induced pluripotent stem cells (human IPSCs), neuronal differentiation

## Abstract

Trace amine-associated receptors (TAARs), which were discovered only in 2001, are known to be involved in the regulation of a spectrum of neuronal processes and may play a role in the pathogenesis of a number of neuropsychiatric diseases, such as schizophrenia and others. We have previously shown that TAARs also have interconnections with the regulation of neurogenesis and, in particular, with the neurogenesis of dopamine neurons, but the exact mechanisms of this are still unknown. In our work we analyzed the expression of TAARs (TAAR1, TAAR2, TAAR5, TAAR6, TAAR8 and TAAR9) in cells from the human substantia nigra and ventral tegmental areas and in human pluripotent stem cells at consecutive stages of their differentiation to dopaminergic neurons, using RNA sequencing data from open databases, and TaqMan PCR data from the differentiation of human induced pluripotent stem cells in vitro. Detectable levels of TAARs expression were found in cells at the pluripotent stages, and the dynamic of their expression had a trend of increasing with the differentiation and maturation of dopamine neurons. The expression of several TAAR types (particularly TAAR5) was also found in human dopaminergic neuron-enriched zones in the midbrain. This is the first evidence of TAARs expression during neuronal differentiation, which can help to approach an understanding of the role of TAARs in neurogenesis.

## 1. Introduction

Trace amines (TA) are a group of biogenic amines, that can be found in mammals in extremely low concentrations, that were long considered as neuromodulators of classical monoaminergic systems [1,2]. Their receptors, known as trace amine-associated receptors (TAARs), which belong to the family of G protein-coupled receptors, were discovered only in 2001 [3,4]. Between 15 and 17 types of TAARs have been described in rodents, while humans have only six functional ones, including TAAR1, TAAR2, TAAR5, TAAR6, TAAR8 and TAAR9 [5].

Expression of all TAARs, except *TAAR1*, were found in the olfactory epithelium [6], and initially these TAARs were considered as receptors that have only olfactory functions [7]. But later it was found that their distribution and functions are much wider.

TAAR1 acts as a modulator of the dopaminergic system [6,8,9]. Changes in the TA and TAAR systems have been demonstrated in several neuropsychiatric diseases, such as schizophrenia [5,10,11,12], depression [11], attention deficit hyperactivity disorder [13], drug addiction [13] and others. Expression of *TAAR1* was identified in brain structures associated with monoaminergic systems [6], in the limbic system, dorsal raphe nucleus [9], prefrontal cortex [14,15], hippocampus [16], spinal cord [3,17] and outside the central nervous system [18,19,20,21]. In our laboratory, it was shown that *TAAR2* is expressed in the piriform cortex, hypothalamus and lateral habenula, as well as in the midbrain and raphe nuclei [22]. Expression of *TAAR5* was found in the limbic structures such as the amygdala, nucleus accumbens, thalamus, hypothalamic nucleus, entorhinal and piriform cortex [23,24]. 

Lately, it was found that the TA and TAAR system is implicated in the regulation of neurogenesis, including adult neurogenesis [22,23,24]. We have shown that TAARs are expressed in cells in zones of adult neurogenesis, in particular in the hippocampus and subventricular zone [25]. Also, we have discovered that animals lacking *TAAR2* gene have significant changes in the dopaminergic system and neurogenic areas. TAAR2 knockouts are characterized by an increased number of dopaminergic neurons in the substantia nigra (SN) and doublecortin-positive proliferating cell nuclear antigen (PCNA)-positive neural progenitor cells in the subventricular zone (SVZ) and subgranular zone (SGZ) of the dentate gyrus (DG). They also have higher levels of dopamine and an elevated expression brain-derived neurotrophic factor (BDNF) in the striatum, and an elevated expression of monoamine oxidase B (MAO-B) in the midbrain [22]. Animals lacking another type of TAAR, the *TAAR5* gene, also have an increased number of dopaminergic neurons in the SN and doublecortin-positive PCNA-positive neural progenitor cells in the SVZ and SGZ. Biochemically, they have significantly elevated levels of dopamine and its metabolites, 3,4-dihydoxyphenylacetic acid (DOPAC) and homovanillic acid (HVA), in the striatum, and we also showed that they have an increased expression of glial cell line-derived neurotrophic factor (GDNF) in the striatum [23]. 

Together, these data indicate that the TA and TAAR system can influence neurogenesis and, in particular, the neurogenesis of dopamine (DA) neurons. And as a first step in understanding the nature of this association, we investigated the expression of TAARs at different stages of the differentiation of human induced pluripotent stem cells (IPSCs) into DA neurons in vitro, as well as in DA-enriched midbrain zones, like the SN and the ventral tegmental area (VTA). 

## 2. Results

### 2.1. TAARs Are Expressed at Low Levels in a Number of Human IPSC Lines, but Not in H9 or MEL1 Embryonic Stem Cell (ESC) Lines

**Figure 1:**
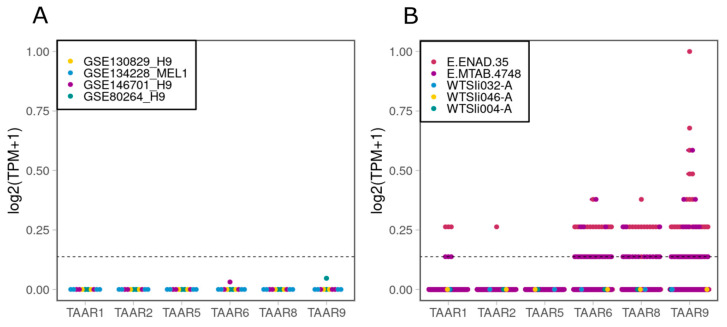
Expression of TAARs in datasets from open databases. (**A**) Datasets GSE130829, GSE134228, GSE146701, GSE 80264 from human ESCs. (**B**) Datasets E.ENAD.35, E.MTAB.4748 and datasets from WTSli004-A, WTSli032-A and WTSli046-A human IPSC lines. The cut-off value of 0.1 TPM is identified by the dotted line.

To understand the dynamics of TAAR expression during the differentiation of pluripotent stem cells to DA neurons, first of all, we analyzed their expression at the stage of pluripotency, using data from human ESCs and IPSCs. As TAARs are characterized by very low levels of expression, all chosen transcriptome datasets in this study had a large number of reads per sample (35 read millions or above per sample). We chose two lines of human ESCs: H9 (WiCell Research Institute) and MEL1 (Stem Cell Sciences plc), as two of the most popular lines, and selected four datasets of their expression profiles from the GEO repository [26,27,28]. We found no expression of any types of TAARs in the chosen datasets (Figure 1A). 

Next, we analyzed the expression profiles from human IPSC lines in five datasets, including the open access RNA-sequencing data for cell lines, which we use in the present study available on the EBiSC, and two datasets for the human IPSCs generated by the Human Induced Pluripotent Stem Cell Initiative (HipSci) project [29], available at the Expression Atlas database. We showed that a low expression of *TAAR6*, *TAAR8*, *TAAR9*, and to lesser extent *TAAR1* and *TAAR2*, could be found in human IPSCs, but the presence and the level of expression varied between cell lines (Figure 1B).

**Figure 2:**
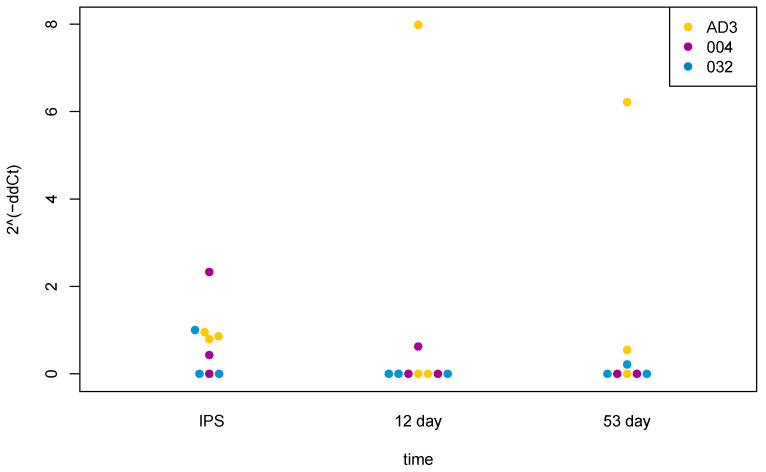
Expression of *TAAR5* at different time points of differentiation of AD3, WTSIi004-A (004), WTSIi032-A (032) lines of human IPSCs to DA neurons, analyzed by TaqMan Realtime PCR. Points represent biological replicates from three human IPSC lines.

Then, we differentiated three human IPSC lines (AD3, WTSIi004-A, WTSIi032-A) to DA neurons by the protocol described previously [30] and analyzed the TAARs’ expression using TaqMan Realtime PCR in various time points of differentiation. We found only sporadic expression of *TAAR5* (Figure 2) in analyzed cell lines at any point of the differentiation process and did not find the expression of any other types of TAARs in our samples.

### 2.2. TAAR5, TAAR6 and TAAR8 Are Expressed in Cells, Isolated from Human SN and VTA

**Figure 3:**
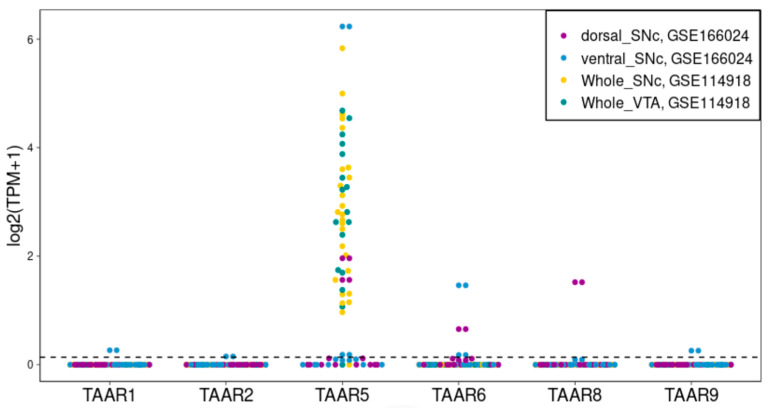
Expression of different types of TAARs in cells, isolated from human SN and VTA, obtained by analysis of high throughput sequencing data, selected from open databases (datasets GSE114918, GSE166024). The cut-off value 0.1 TPM is identified by the dotted line.

We estimated the TAAR expression in SN and VTA isolated from the human brain by the analysis of two high throughput sequencing-generated datasets, GSE114918 and GSE166024, represented in the GEO repository [31,32]. The data show a meaningful expression of *TAAR5* in neurons from both analyzed areas (Figure 3) and a lack of expression of any other types of TAARs in the studied structures.

### 2.3. TAARs’ Profiles of Expression Are Changed during Differentiation of Human Pluripotent Stem Cells to Midbrain Neurons

**Figure 4:**
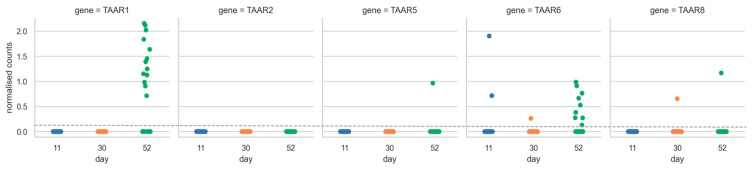
Expression of TAARs in samples from day 11, 30 and 52 of differentiation of human IPSCs to midbrain neurons (dataset EGAD0000100615). Each point represents a single-cell expression from 215 mixed cell lines.

We analyzed the dataset EGAD00001006157, which was obtained from the European Genome-phenome Archive. It contains RNA sequencing data from 215 human IPSC lines, which were mixed and differentiated towards midbrain neurons, and obtained cell culture contained dopaminergic neurons [33]. The differentiation protocol was based on the protocol published by Kriks et al., 2011 [34]. Samples for RNA sequencing were collected on days 11, 30 and 52 of differentiation. We found a single observation of *TAAR6* expression on day 11 of differentiation, while the expression of other TAARs was absent, and this picture remained stable till day 30 of differentiation (Figure 4). By day 52 of differentiation, we observed significant expression levels of *TAAR1* and *TAAR6*, as well as single observations of expression of *TAAR8* and *TAAR5*.

**Figure 5:**
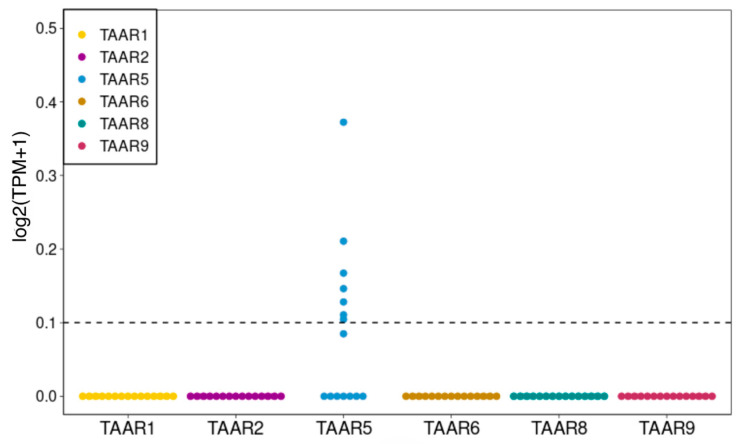
Expression of TAARs on day 16 of differentiation of human ES cells (dataset GSE86654). The cut-off value 0.1 TPM is identified by the dotted line.

The dataset GSE86654 [35] contains single-cell RNA sequencing data obtained from dopaminergic progenitors on day 16 of the differentiation of ESCs (H9 cell line), made using the protocol described in Nolbrant S. et al., 2017 [36] (Figure 5). Sporadic *TAAR5* expression was identified in these cells, but any other TAAR expression was absent. 

**Figure 6:**
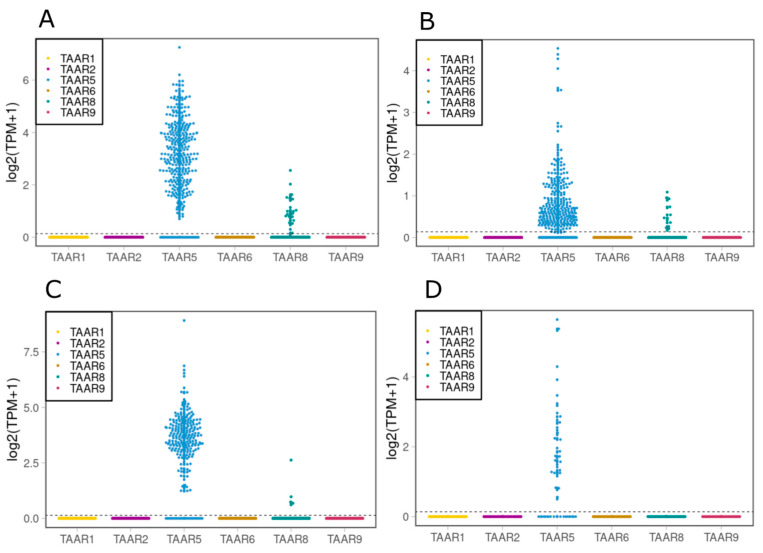
Expression of TAARs in dopaminergic progenitors and in cells obtained from human fetal midbrain, analyzed by single cell RNA sequencing (dataset GSE118412). (**A**) Dopaminergic progenitors on day 16 of differentiation using protocol published in Nolbrant et al., 2017. (**B**) Cells obtained from dopaminergic progenitors after 6 months of transplantation to striatum of 6-OHDA rats. (**C**) Cells obtained from human fetal midbrain. (**D**) Cells obtained from human fetal midbrain after 6 months of transplantation to striatum of 6-OHDA rats. The cut-off value of 0.1 TPM is identified by the dotted line.

Next, we took for analysis the dataset GSE118412 [37]. This dataset contains single-cell RNA sequencing data from DA neuronal precursors obtained on day 16 of differentiation from human ESCs (RC17 cell line), made using the protocol described in Nolbrant S. et al., 2017 [36], or from human fetal ventral midbrain (fMB) cells (Figure 6). Both types of cells were analyzed under two conditions: before the transplantation into the striatum of 6-OHDA rats, and after 6 months of transplantation [37].

We showed that both progenitors of DA neurons on day 16 of differentiation, and in grafts from human fMB, had a significant level of expression of *TAAR5* and *TAAR8*. After 6 months of transplantation this remained stable with a small fall in the number of cells obtained from dopaminergic progenitors which had *TAAR8* expression; however, cells from human fMB lacked expression of *TAAR8* at all. 

We compared the results of the analysis performed on the data from the open sources with data obtained by TaqMan Realtime PCR during the differentiation of human IPSC lines AD3, WTSli004-A and WTSli032-A into DA neurons, made using protocol described previously [30] (Figure 2), where samples for RNA isolation were collected from undifferentiated human IPSCs, on days 12 and 53. Using TaqMan Realtime PCR, we did not find any expression levels of *TAAR1*, *TAAR2*, *TAAR6*, *TAAR8* or *TAAR9* at any of the stages of differentiation of human IPSCs into dopaminergic neurons. But as we already mentioned, we observed *TAAR5* expression at a low level in undifferentiated cells, especially in the WTSli004-A cell line, and it was higher in a small number of samples on day 12 and day 53 of differentiation; however, there were only sporadic observations in biological replicates, and changes in expression were not significant statistically for any of the observed cell lines (following one-way ANOVA analysis).

## 3. Discussion

TAARs were first discovered in 2001 [3,4], and later, the majority of them were characterized as olfactory receptors, detecting innate odors encoded by the endogenous or exogenous (bacterial) decarboxylation of amino acids [7]. However, it is clear now that their functions and distribution are much wider, but the full spectrum of processes in which they play roles is still poorly understood [1,2]. 

The specific regulatory role of TAARs was found in various physiological processes, such as attention, mood, nutrition and movement. Also, it is known that changes in the TA and TAAR system might contribute to the pathogenesis of several mental diseases, such as schizophrenia, depression, drug addiction and others [10,11,38]. The interaction of TAARs with the regulation of neurogenesis and neurogenesis of DA neurons was shown previously using TAAR knockout animals [22,23,24]. It was found that *TAAR2* and *TAAR5* are expressed in neurogenic zones, such as the SVZ and SGZ [25], and knockout animals lacking TAAR2 or TAAR5 have significant changes in the dopaminergic system and neurogenesis [22,23,24].

To understand the role of TAARs in the process of development and maturation of dopaminergic neurons, we used open access RNA sequencing data to analyze the expression of TAARs in human pluripotent stem cells, at different stages of differentiation, and in mature neurons in the SN and VTA in the midbrain. We found no expression over the threshold of any type of TAARs in human ESCs during the examination of RNA sequencing data from two frequently used cell lines, RC17 and MEL1. We obtained the same results with three human IPSC lines—WTSIi004-A, WTSIi032-A and WTSIi046-A, but the analysis of human IPSCs HipSci cohort, provided in the E.ENAD.35 and E.MTAB.4748 datasets, showed that the expression of *TAAR1*, *TAAR2*, *TAAR6*, *TAAR8* and especially *TAAR9* can be detected in cells at pluripotent stage. Thus, the expression of TAARs at the pluripotent stage seems to be various and cell line-dependent.

We showed the presence of the expression of TAARs in mature neurons from areas specific to DA neurons in the midbrain, i.e., the SN and VTA. We found that cells from these areas in humans have a stable and meaningful expression of *TAAR5*, against the lack of expression of any other TAARs. The discovery of TAARs in areas that are associated with DA neurons could be expected, since the TA and dopaminergic systems are generally strongly connected. Before the discovery of TAARs, it was believed that TAs generally acted indirectly on DA neurons through interaction with the dopamine transporter, but recent data show that the signaling cascades of these two neurotransmitter networks are greatly overlapped, including direct interaction between their receptors [1,3].

Analysis of TAAR expression at the distinct stages of differentiation was performed on the RNA sequencing data from the open databases and the TaqMan Realtime PCR results, obtained during the differentiation of three human IPSC lines to dopaminergic neurons in vitro. These data indicate the tendency of the expression of TAARs to increase in more differentiated stages. The examination of data from nonspecific midbrain differentiation showed that TAAR expression, which was only sporadic on day 11, escalated until day 53, when the expression of *TAAR1* and *TAAR6* become significant. 

A considerable expression of *TAAR5* was observed on day 16 of human IPSC differentiation to DA neurons, which was made using a protocol published in Nolbrant S. et al., 2017 [36]. These observations were confirmed by the analysis of another dataset, which contains RNA sequencing data from DA neuronal precursors and cells from human fetal midbrain, which were analyzed before and after transplantation to the striatum of 6-OHDA rats. These data showed that expression of *TAAR5* and *TAAR8* could be found in both of the analyzed cell types, before and after transplantation, with a partial difference between these two cell types. These observations are notably consistent with the results obtained from the mature cells of SN and VTA.

We should indicate that our study has some limitations, since we analyzed data from cells during differentiation of human IPSCs, which was performed using different protocols, and therefore the characteristics of the resulting cells may differ depending on the used protocol. 

Thus, we have shown that TAARs have various and cell line-dependent expression levels in human pluripotent stem cells. TAARs are expressed during various periods of differentiation of dopaminergic neurons and the dynamic of their expression has an increasing trend in particular, *TAAR5*, *TAAR8*, *TAAR6* and *TAAR1* (with less specific differentiation), in more mature neurons. This process probably depends on the cell type and the protocol of differentiation. Also, we documented that *TAAR5* is expressed in the cells from the SN and VTA in the midbrain.

Overall, the obtained data expand the knowledge of TAAR expression distribution in the various cell types in the distinct differentiation stages, but do not yet answer the question about the role of TAARs in the development of DA neurons. This important topic remains open for further research, where first of all it would be necessary to evaluate the impact of TAARs’ knockout on the neuronal differentiation.

## 4. Materials and Methods

### 4.1. Public Transcriptomic Data Analysis

The expression data for stem cells, DA neurons and the dopaminergic midbrain structures were derived from the Gene Expression Omnibus (GEO) repository [39]. The selected datasets are listed in Table 1. Additionally, we included the RNA-sequencing data for human IPSCs from the human pluripotent stem cell registry (hPSCreg) [40] and Expression Atlas [41]. 

Gene expression data were normalized to transcript per million (TPM). The value 0.1 PM was applied as the threshold for positive expression. Data were visualized using the ggplot2 r package [42,43,44,45,46].

### 4.2. Cell Lines

Human IPSC lines were cultivated under standard conditions (CO_2_ 5%, O_2_ 20%, temperature 37 °C). Cells were maintained under feeder-free conditions on Geltrex (Thermo Fisher Scientific, A1413202, Paisley, UK) coated dishes in mTeSR1 Basal Medium (STEMCELL) with the addition of Penicillin-Streptomycin 1× (Thermo Fisher Scientific). ReLeSR (STEMCELL, Catalog #05872, Vancouver, BC, Canada) was used for cell dissociation.

#### Human IPSC Lines

- The human IPSC line (AD3) was generated from human newborn fibroblasts (HNFs) using the lentiviral nonintegrating Sendai reprogramming kit (CytoTune-iPS 2.0 Sendai Reprogramming kit (Invitrogen, Paisley, UK) according to the manufacturer’s instructions. HNFs were purchased from Lonza and were cultured as described [47]. The generated human IPSCs were cultured under feeder-free conditions and maintained on plates coated with Matrigel (growth factor reduced; BD, USA) with mTeSR1 (STEMCELL) at 37 °C, 5% CO_2_ and 21% O_2_ according to WiCell Inc. protocols. Cells were passaged every 4–5 days at ∼80% confluence by using 0.02% EDTA (Versene). A generated AD3 Sendai-derived human IPSC line was characterized according to the protocol published before [48] and fulfilled all pluripotency criteria [47];
- WTSIi004-A (HPSI1113i-qolg_3) from the European Bank for Induced pluripotent Stem Cells (EBiSC), Biosample ID SAMEA2464810
- WTSIi032-A (HPSI1113i-bima_1) from EBiSC, Biosamples ID SAMEA2399246

The EBiSC Bank acknowledges the Wellcome Trust Sanger Institute (WTSI) as the source of the human induced pluripotent cell lines WTSIi032-A (HPSI1113i-bima_1) and WTSIi004-A (HPSI1113i-qolg_3), which were generated with support from EFPIA companies and the European Union (IMI-JU’).

### 4.3. Differentiation of Human IPSCs

Differentiation of human IPSCs was performed according to the protocol, which was previously described by [30]. Briefly, for differentiation, human IPSCs were harvested with TrypLE Select Enzyme (Gibco, Paisley, UK) and seeded into 6-well plates coated with Geltrex/Knockout DMEM, at a density of 4 × 10^5^ cells per well. During seeding, 5 μM Y27632 was added to the medium. On the third day after seeding, cells were transferred to xGMEM medium (Gibco), containing 8% Knockout serum replacement (Thermo), 0.1 mM NEAA (Gibco), 0.1 mM beta-mercaptoethanol, 1 mM Pyruvate (Gibco), 1% Penicillin-Streptomycin, 2 mM L-glutamine supplemented with 500 nM A83-01 (STEMCELL, Vancouver, BC, Canada) and 2 µM Purmorphamine (Sigma, Roedermark, Germany), as well as 100 ng/mL FGF8b (STEMCELL) from days 1 to 7, 100 nM LDN193189 (STEMCELL) from days 1 to 12 and 3 μM CHIR99021 from days 3 to 12. The medium was changed daily.

On day 12 of differentiation, cells were harvested with Accumax (STEMCELL) and plated on 24-well AggreWell-800 plates (STEMCELL), at a density of 1.2 million cells per well, according to the manufacturer’s protocol, in a medium containing Neurobasal medium (Gibco), 1% Penicillin-Streptomycin, 0.1 mM beta-mercaptoethanol, 200 µM ascorbic acid (Sigma), 2 mM L-glutamine (Gibco), 400 µM dbc AMP (STEMCELL) and B-27 supplement 1× (Gibco), supplemented with 10 ng/mL GDNF (STEMCELL) and 20 ng/mL BDNF (STEMCELL) for the formation of embryonic bodies. During seeding, Y27632 5 μM was added to the medium. The medium was changed the day after seeding and then every 3 days.

On the day 28 of differentiation, the embryonic bodies were dissociated using Accumax (STEMCELL). The resulting cell suspension was used for the final differentiation and maturation under in vitro conditions. Cells were seeded into 96-well plates coated with Geltrex/Neurobasal medium (Gibco), at a density of 7.5 × 10^4^ cells per well, in Neurobasal medium (Gibco), 1% Penicillin-Streptomycin, 0.1 mM beta-mercaptoethanol, 200 μM ascorbic acid (Sigma), 2 mM L-glutamine (Gibco), 400 µM dbc AMP (STEMCELL) and B-27 supplement 1× (Gibco), supplemented with 10 ng/mL GDNF (STEMCELL) and 20 ng/mL BDNF (STEMCELL). During seeding, Y27632 5 μM was added to the medium. The medium was changed the next day after passage and then every 3 days. Cells were cultured for up to 53 days.

### 4.4. RNA Isolation and Reverse Transcription

Cells were harvested for RNA isolation on days 0, 12 and 53 of differentiation. Three biological replicates were used for each cell line. RNA was isolated from cells using TRIzol (Invitrogen). For reverse transcription, 1 µg of RNA was used. TURBO DNase (Thermo Fisher Scientific) was used to purify samples from residual genomic DNA. Reverse transcription was performed using the RevertAid reverse transcriptase (ThermoFisher Scientific). All procedures were performed in accordance with the manufacturer’s recommendations. 

### 4.5. TaqMan Real-Time PCR

A TaqMan Realtime PCR assay was used to assess the TAARs’ mRNA expression levels. TaqMan Realtime PCR was performed using the qPCRmix-HS kit (Eurogen, Moscow, Russia) with the QuantStudio™ 5 Real-Time PCR System (Applied Biosystems, Waltham, MA, USA) with the following primers and probes (Table 2). Relative expression quantification was performed using the 2^−ΔΔCt^ method, representing the expression of the gene of interest relative to the expression of the housekeeping gene. During the analysis, the following parameters were calculated for each reaction: Ct value (ΔCt), ΔCt value (ΔΔCt) for replicates, 2^−ΔΔCt^ value and the fold ratio of expression levels in samples (for the comparison of the individual experimental groups with the average value for an internal control sample). The level of expression of the studied gene was normalized according to the level of expression of the housekeeping gene S18. A one-way ANOVA test was used for statistical analysis.

## Figures and Tables

**Table 1:** Analyzed datasets.

Dataset ID	Title	Group Description	*n*
GSE80264 [26]	Transcriptome analysis of H9 human ESC-derived cerebral organoids	H9 ESC line	2
GSE130829	Effect of cell division on cell differentiation (RNA-Seq H9 cells UD E24 E24HU E24Noc)	H9 ESC line	2
GSE146701 [27]	Regulation of histone H3 by the ubiquitin-conjugating enzyme UBE2K determines neurogenesis of human ESCs [RNA-Seq]	H9 ESC line	3
GSE134228 [28]	In vitro pancreas differentiation of human ESCs line Mel1.	Mel1 ESC line	4
E-ENAD-35	HipSci Project—RNA-seq of healthy volunteers	Human IPSCs derived from healthy individuals	191
E-EMTAB-4748	RNA-seq of coding RNA in human fibroblasts, peripheral blood mononuclear cells (PBMCs) and IPSCs as part of the HipSci project	IPSCs derived from healthy individuals	187
GSE166024 [31]	Human-specific transcriptome of ventral and dorsal tiers of the SN pars compacta dopamine neurons	Dorsal midbrain dopamine neurons	7
		Ventral midbrain dopamine neurons	7
GSE114918 [32]	RNA-seq of human SNc and VTA midbrain dopamine neurons isolated from post-mortem material of control subjects and Parkinson’s Disease patients using laser capture microdissection	Substantia nigra	25
		Ventral tegmental area	16
GSE86654 [35]	Identifying markers predicting successful graft outcome for clinical translation of human ESC-based cell therapy for Parkinson’s disease	Human ESCs differentiated to ventral midbrain progenitors (high DA group)	9
		Human ESCs differentiated to ventral midbrain progenitors (low DA group)	6
GSE118412 [37]	Single cell transcriptomics identifies stem cell-derived graft composition in a model of Parkinson’s disease	Dopaminergic progenitors at day 16 of differentiation, obtained from RC17human IPSC line	404
		Dopaminergic progenitors at day 16 of differentiation, obtained from RC17 human IPSC line 6 months after transplantation to striatum of 6-OHDA rats	683
		Cells from human fetal midbrain	256
		Cells from human fetal midbrain 6 months after transplantation to striatum of 6-OHDA rats	63
EGAD00001006157 [33]	Single cell RNA sequencing data from differentiation of 215 cell lines of human IPSCs to midbrain fate, including dopaminergic neurons	Midbrain fate neurons	42

**Table 2:** Primers used.

Gene	Type	Sequence	Location
TAAR1	Forward	tgaccacactcgttggcaatctg	283–305
	Reverse	acagtgctcagcagatctcacca	421–443
	Probe	FAM-ggccactgtggactttcttctggggt-BHQ1	374–399
TAAR2	Forward	ggtccctggagcatttgccttc	507–528
	Reverse	gccataaacaaggtggtccccc	626–647
	Probe	FAM-tggttgcttgttccagttcctgccca	581–606
TAAR5	Forward	agcaccattcgctcagtggaga	323–341
	Reverse	gtgaggcagaagagggtgtcca	369–388
	Probe	FAM-ttcctctgccgcctgcacacct	364–386
TAAR6	Forward	atgtacagcggtgctgtgtt	391–411
	Reverse	caacggtctgacaacctcct	561–580
	Probe	FAM-ggctggaggaattatctgatgcc-BHQ1	527–549
TAAR8	Forward	acacaggtgtcaatgatgatggg	512–534
	Reverse	atttgacagccacctacgca	562–581
	Probe	ctggaggaattagtaagtgctctc	535–558
TAAR9	Forward	gccaggctccactgaatcaa	611–630
	Reverse	agcttggctggctgtacttt	697–716
	Probe	FAM-ggtggccaagcatcaggctaggaa	666–689
HPRT	Forward	ggctccgttatggcgacc	139–156
	Reverse	tcgagcaagacgttcagtcc	281–300
	Probe	FAM-cagccctggcgtcgtgattagtg	159–181
RPS18	Forward	tcaacaccaacatcgatgggcg	92–113
	Reverse	gctttcctcaacaccacatgagca	165–188
	Probe	FAM-actgccattaagggtgtgggccga	136–159

## Data Availability

Publicly available datasets were analyzed in this study. Used datasets are GSE80264 [26], GSE130829, GSE146701 [27], GSE134228 [28], E-ENAD-35, E-EMTAB-4748, GSE166024 [31], GSE114918 [32], GSE86654 [35], GSE118412 [37], EGAD00001006157 [33].

## Abbreviations

TA	Trace Amines
TAARs	Trace Amine-Associated Receptors
SN	Substantia Nigra
PCNA	Proliferating Cell Nuclear Antigen
SVZ	Subventricular Zone
SGZ	Subgranular Zone
DG	Dentate Gyrus
BDNF	Brain-Derived Neurotrophic Factor
MAO-B	Monoamine Oxidase B
DOPAC	3,4-Dihydoxyphenylacetic Acid
HVA	Homovanillic Acid
GDNF	Glial Cell Line-Derived Neurotrophic Factor
DA	Dopamine
IPSC	Induced Pluripotent Stem Cell
fMB	Fetal Ventral Midbrain
ESC	Embryonic Stem Cell
